# Immortalizing Mesenchymal Stromal Cells from Aged Donors While Keeping Their Essential Features

**DOI:** 10.1155/2020/5726947

**Published:** 2020-06-16

**Authors:** María Piñeiro-Ramil, Rocío Castro-Viñuelas, Clara Sanjurjo-Rodríguez, Silvia Rodríguez-Fernández, Tamara Hermida-Gómez, Francisco J. Blanco-García, Isaac Fuentes-Boquete, Silvia Díaz-Prado

**Affiliations:** ^1^Grupo de Investigación en Terapia Celular y Medicina Regenerativa, Departamento de Fisioterapia, Medicina y Ciencias Biomédicas, Facultad de Ciencias de la Salud, Universidade da Coruña (UDC), Instituto de Investigación Biomédica de A Coruña (INIBIC), Complexo Hospitalario Universitario de A Coruña (CHUAC) Servizo Galego de Saúde (SERGAS), Galicia, Spain; ^2^Centro de Investigaciones Científicas Avanzadas (CICA), Agrupación estratégica CICA-INIBIC, Universidade da Coruña, Galicia, Spain; ^3^Centro de Investigación Biomédica en Red de Bioingeniería Biomateriales y Nanomedicina (CIBER-BBN), Spain; ^4^Grupo de Investigación en Reumatología (GIR), Instituto de Investigación Biomédica de A Coruña (INIBIC), Complexo Hospitalario Universitario da Coruña (UDC-CHUAC), Servizo Galego de Saúde (SERGAS), Galicia, Spain

## Abstract

Human bone marrow-derived mesenchymal stromal cells (MSCs) obtained from aged patients are prone to senesce and diminish their differentiation potential, therefore limiting their usefulness for osteochondral regenerative medicine approaches or to study age-related diseases, such as osteoarthiritis (OA). MSCs can be transduced with immortalizing genes to overcome this limitation, but transduction of primary slow-dividing cells has proven to be challenging. Methods for enhancing transduction efficiency (such as spinoculation, chemical adjuvants, or transgene expression inductors) can be used, but several parameters must be adapted for each transduction system. In order to develop a transduction method suitable for the immortalization of MSCs from aged donors, we used a spinoculation method. Incubation parameters of packaging cells, speed and time of centrifugation, and valproic acid concentration to induce transgene expression have been adjusted. In this way, four immortalized MSC lines (iMSC#6, iMSC#8, iMSC#9, and iMSC#10) were generated. These immortalized MSCs (iMSCs) were capable of bypassing senescence and proliferating at a higher rate than primary MSCs. Characterization of iMSCs showed that these cells kept the expression of mesenchymal surface markers and were able to differentiate towards osteoblasts, adipocytes, and chondrocytes. Nevertheless, alterations in the CD105 expression and a switch of cell fate-commitment towards the osteogenic lineage have been noticed. In conclusion, the developed transduction method is suitable for the immortalization of MSCs derived from aged donors. The generated iMSC lines maintain essential mesenchymal features and are expected to be useful tools for the bone and cartilage regenerative medicine research.

## 1. Introduction

Human bone marrow-derived mesenchymal stromal cells (MSCs) are a promising cell source for bone and cartilage therapies due to their self-renewal capacity and multipotency [1–4]. However, culture-expanded MSCs progressively lose these capacities, which is a major limitation for research [2–5]. Moreover, both proliferative and differentiation potentials of MSCs decrease with donor aging [3, 6, 7]. As a result, research involving MSCs derived from aged donors is limited by both *in vitro* expansion-induced senescence and donor-related reduction of proliferation. This is a major bottleneck for research on MSC-based regeneration of skeletal tissues in age-related chronic joint diseases, with osteoarthritis (OA) being one of the most common and disabling ones [3, 8].

This proneness to senescence of aged MSCs may be overcome by immortalization, which requires repression of p53- and Rb-mediated pathways and telomere maintenance. Cell immortalization can be achieved by either transduction of immortalizing genes like simian virus 40 large T antigen (SV40LT) [9, 10] or human papillomavirus E6/E7 gene [11] which promote cell cycle progression, or human telomerase reverse transcriptase (hTERT) which prevents telomeres shortening [12–14]. Transduction of single SV40LT/E6/E7 or hTERT can fail to immortalize primary human cells [15, 16] and specifically MSCs [14, 17–19], while the combination of SV40LT and hTERT has been shown to be useful for generating immortalized MSC lines [17]. Nevertheless, most immortalized MSC lines have been generated from healthy and/or young donors [9, 11, 12, 14], whereas “aged” and “diseased” MSC lines are still lacking. The underlying cause might be that retroviral transduction is limited by their inefficiency in infecting aged and/or diseased donor-derived MSCs because they are slow-dividing cells [20].

Therefore, strategies to enhance infection efficiency should be employed. One of these strategies is spinoculation, which has been used during decades to improve viral infection of several types of cells *in vitro* [21–34] (Table 1), although the process responsible for spinoculation-induced enhancement of infection has not been discovered yet [33]. However, it is known that the enhancement of infection induced by spinoculation is cell type-dependent [20, 25] and also related to the speed of centrifugation [33] in a cell type-dependent manner [23]. Therefore, spinoculation parameters must be optimized for each transduction system (virus and target cell type). Since spinoculation-induced enhancement of infection is also related to virus concentration, it could be possible to increase it by prolonging the posttransfection incubation of packaging cells before harvesting [22, 28, 32, 33]. As virus half-life at 37°C is shorter than at 32°C, packaging cell incubation and centrifugal infection may need to be performed at 32°C [21, 27–29].

For greater enhancement of infection, chemical adjuvants for transduction and inductors of transgene expression can be used. Hexadimethrine bromide (HDMB, also known as *Polybrene*) is the most usual adjuvant for retroviral infection but should be used over short application times and at low concentrations due to the high cellular toxicity [22, 35]. The combination of HDMB and spinoculation may have a synergistic effect in enhancing infection [36], but it has been proposed that centrifugation-induced stress produced during spinoculation also might increase cell susceptibility to HDMB toxicity [35].

The histone deacetylase inhibitor valproic acid (VPA, 2-propyl-valeric acid) could be used for transgene expression induction [37, 38]. VPA not only prevents the transcriptional silencing of genes [39] but also reduces cell proliferation [39, 40] and might even exert a negative [39] or positive effect on cell viability, depending on the concentration and cell type [37, 40]. Thus, the concentration needs to be adjusted in order to enhance transgene expression without affecting cell viability and proliferation.

Taking all this information into account, the aim of this study was to develop an efficient method for immortalization of slow-dividing cells, like MSCs from aged donors, without losing the parental phenotype.

## 2. Materials and Methods

### 2.1. Isolation and Culture of Human Bone Marrow-Derived Mesenchymal Stromal Cells (MSCs)

**Figure 1:**
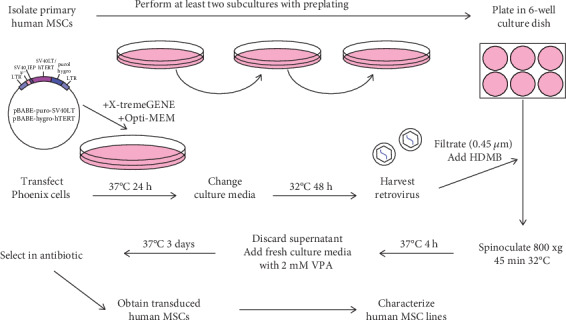
Human MSC immortalization method, including SV40LT and hTERT retroviruses produced by Phoenix cells, spinoculation with HDMB, and transgene expression induction by VPA.

The present study was reviewed and approved by the Ethics Committee of Research from A Coruña-Ferrol, Spain (2016/588). The bone marrow samples were collected from patients who underwent orthopaedic surgery, after obtaining written informed consent from each donor: eight patients with hip OA (ages ranging from 45 to 94 y.o., five males and three females) and three patients with hip fracture but no OA (ages ranging from 65 to 88 y.o., two males and one female). The bone marrow of femoral heads was washed with Dulbecco's Modified Eagle's Medium (DMEM; Lonza, Madrid, Spain) supplemented with 5% foetal bovine serum (FBS, Gibco, Thermo Fisher Scientific, Madrid, Spain) and 1% penicillin/streptomycin (P/S, Gibco, Thermo Fisher Scientific) (5%FBS/DMEM). After centrifugation at 430 ×*g* for 10 min, cells were plated in adherent culture dishes (Costar Corning Incorporated, New York, USA) and grown in DMEM supplemented with 20% FBS and 1% P/S (20%FBS/DMEM) at 37°C [41]. Subculturing was performed when cell confluence reached 80-90%, including a 15 min preplating technique [42] at the first and second passages (Figure 1). Obtained MSCs were used for spinoculation experiments at the third to fifth passages.

### 2.2. MSC Spinoculation with Retrovirus Produced by Phoenix Cells

Phoenix amphotropic (ATCC® CRL-3213™) cells [43] were plated on 100 mm adherent culture dishes (Costar Corning Incorporated), grown in 10% FBS/DMEM and transfected employing one out of two plasmids, both obtained from Addgene: pBABE-puro-SV40LT (plasmid #13970), deposited by Thomas Roberts [44], or pBABE-hygro-eGFP-hTERT (plasmid #28169), deposited by Kathleen Collins [45]. For each culture dish, 10 *μ*g of plasmid were mixed with Opti-MEM (Gibco, Thermo Fisher Scientific) up to a volume of 970 *μ*l. Then, 30 *μ*l of X-tremeGENE HP DNA Transfection Reagent (Roche, Sigma-Aldrich Química S.A.) were added, and this mixture was incubated for 25 minutes at room temperature. Thereafter, the mixture was added to culture dishes in a dropwise manner. Transfected Phoenix cells were incubated at 37°C during 24 hours, whereupon the culture medium was changed and cells were incubated at 32°C for retrovirus production [21, 29].

After incubation at 32°C, the supernatant was collected and filtered through a 0.45 *μ*m pore size membrane filter (Millipore, Burlington, Massachusetts, USA). 8 *μ*g of HDMB (Sigma-Aldrich Química S.A.) was added per ml of retroviral supernatants [28, 46], and this mixture was employed for the infection of MSCs at 70% confluence in 6-well adherent culture dishes (Costar Corning Incorporated) (Figure 1). No viruses were added to one well of each dish, which was used as a negative control for antibiotic selection.

Different MSC spinoculation experiments were performed, including coinfections with SV40LT and hTERT retroviruses as well as infections with single SV40LT or hTERT viruses. A volume of 1.5 ml retroviral supernatant was added to each well. Two centrifugation speeds (1000 and 800 ×*g*) and three time-points (60, 45, and 30 minutes) were assayed for spinoculation (Table 2), which was performed at 32°C. After spinoculation, MSCs were incubated for 4 hours at 37°C, and after, retroviral supernatants were replaced by a fresh culture medium with different concentrations of VPA (Cayman Chemical Company, Ann Arbor, Michigan, USA) [37] (Table 2). After three days, the culture medium was replaced by a selection culture medium containing 2.5 *μ*g/ml puromycin (Thermo Fisher Scientific) or 75 *μ*g/ml hygromycin (AMRESCO, VWR International, Radnor, Pennsylvania, USA) (Figure 1).

### 2.3. Optimization of VPA Concentration

VPA was added to the culture medium after retroviral infection to induce transgene expression. To determine the optimal VPA concentration, cell viability measurements were carried out in three MSC transduction experiments with SV40LT retrovirus. Cell viability was determined by employing Cell Counting Kit-8 (CCK-8) (Sigma-Aldrich Química S.A.) (1) before spinoculation; (2) after spinoculation, just before VPA addition; (3) after three-day incubation with a culture medium containing 0.5, 1, 2, 3, or 5 mM VPA; and (4) after selection of transduced cells in a culture medium containing 2.5 *μ*g/ml puromycin. Absorbance measurements were performed in triplicate using a NanoQuant Infinite M200 microplate reader (Tecan Ibérica Instrumentación S.L., Barcelona, Spain) with a measurement wavelength of 450 nm and a reference wavelength of 650 nm, following the manufacturer's instructions.

### 2.4. Testing SV40LT and eGFP-hTERT Nuclear Expression in Transduced MSCs

Transduced MSCs seeded in 8-well chamber slides (Millipore) were washed with phosphate-buffered saline (PBS; Dako, Agilent Technologies Spain S.L., Barcelona, Spain), fixed with 4% paraformaldehyde (Sigma-Aldrich Química S.A.), quenched with 1% glycine, permeabilized with 0.5% Triton X-100 (Sigma-Aldrich Química S.A.), and blocked with 4% bovine serum albumin (Sigma-Aldrich Química S.A.). Subsequent incubation with primary antibodies was performed at 4°C overnight: SV40LT (SV40LT clone Pab 108; 1 : 100; Santa Cruz Biotechnology, Dallas, Texas, USA) and GFP (anti-GFP conjugated to AF488; 1 : 500; A-21311, Invitrogen, Thermo Fisher Scientific).

After three washes in PBS, cells were incubated with a fluorescent goat anti-mouse secondary antibody (A-11032; 1 : 1000; Invitrogen, Thermo Fisher Scientific) at room temperature for one hour. After three additional washes in PBS, two-minute incubation with Hoechst (bisBenzimide H 33342 trihydrochloride, Sigma-Aldrich Química S.A.) was performed. Slides were mounted with Glycergel aqueous mounting medium (Dako, Agilent Technologies Spain S.L.) and observed by employing an Olympus BX61 fluorescence microscope (Olympus Iberia S.A., Barcelona, Spain) coupled to an Olympus DP70 digital camera (Olympus Iberia S.A.). Immunostained SV40LT was identified by red fluorescence while enhanced Green Fluorescent Protein-hTERT (eGFP-hTERT) was identified by green fluorescence. Fluorescence micrographs were obtained employing the cellSens Dimension software (Olympus Iberia S.A.).

### 2.5. Analysis of Morphology and Proliferative Capacity of Transduced MSCs

Cells were observed with a Nikon Eclipse TS100 inverted microscope (Nikon Instruments Europe B.V., Amsterdam, Netherlands) coupled to a XM Full HD digital camera (Hangzhou Xiongmai Technologies (XM), Hangzhou, China). iMSC proliferation was calculated as cumulative population doublings (PDs) at each passage, following the formula in Equation (1), where Nf is the final cell number, Ni is the initial cell number, and log is the natural logarithm [46]. The number of accumulated generations per days in culture was analysed by regression for each cell line. Generation time was calculated for each cell line at each passage as the number of PDs per day, and generation times of all cell lines were compared.

(1) $$\begin{array}{c} \text{PD}=\frac{\text{logNf}‐\text{logNi}}{\log2}, \end{array}$$

Histochemical staining for senescence-associated *β*-galactosidase activity was performed for each cell line after reaching more than 100 PDs at three different passages, using the Senescence Cell Histochemical Staining kit (Sigma-Aldrich Química S.A.). After 16 hours of incubation, *β-*galactosidase-positive and *β-*galactosidase-negative cells were counted on ten random microscope fields and percentage of senescent cells was calculated. Results are provided as mean percentage of senescent cells ± standard error. Senescence values of iMSCs and primary MSCs were compared.

### 2.6. Testing the Preservation of MSC Surface Marker Expression in Transduced MSCs

Mesenchymal stromal cell-positive (CD29, CD44, CD73, CD90, and CD105) and mesenchymal stromal cell-negative (CD34 and CD45) surface marker expression was analysed by flow cytometry in primary and transduced MSCs. For comparison with another MSC line, the expression of the aforementioned surface markers was also analysed in the 3a6 line [47], kindly provided by Dr. Hung's group. Cells were trypsinized, washed twice in Fluorescence Activated Cell Sorting (FACS) buffer (BD Biosciences, Madrid, Spain), and incubated at 4°C for 45 minutes with the following antibodies: fluorescein isothiocyanate (FITC) isotype (clone ICIG1 (1 : 50), Immunostep S.L., Salamanca, Spain), phycoerythrin (PE) isotype (clone B11/6 (1 : 50), Immunostep S.L.) PE/Cy5 isotype (clone 1F8 (1 : 25), Abcam, Cambridge, UK), PE-conjugated anti-human CD29 (clone VJ1/14 (3 : 50), Immunostep S.L.), PE-conjugated anti-human CD34 (clone 581 (2 : 25), BD Pharmingen, Madrid, Spain), FITC-conjugated anti-human CD44 (clone IM7 (1 : 50), BD Pharmingen), FITC-conjugated anti-human CD45 (clone D3/9 (3 : 50), Immunostep S.L.), PE-conjugated anti-human CD73 (clone AD2 (3 : 50) Immunostep S.L.), PE/Cy5-conjugated anti-human CD90 (clone 5E10 (1 : 50) Immunostep S.L.), and FITC-conjugated anti-human CD105 (clone SN6 (1 : 50), AbD Serotec, Bio-Rad Laboratories, S.A., Madrid, Spain). After incubation, cells were washed, resuspended in FACS buffer, and transferred to polypropylene tubes (NUNC, VWR International). Acquisition was made with a BD FACSCalibur flow cytometer (BD Biosciences), and data obtained was analysed using BD CellQuest Pro software (BD Biosciences). For each assay, a minimum of 10^5^ cell events were acquired and analysed.

### 2.7. Testing the Preservation of MSC Multipotency in Transduced MSCs

Primary and transduced MSCs were differentiated towards osteoblasts, adipocytes, and chondrocytes.

#### 2.7.1. Osteogenesis

For osteogenic cell differentiation, two types of experiments were carried out: monolayer differentiation and three-dimensional osteogenesis through endochondral ossification. For monolayer differentiation, 2 × 10^4^ cells were plated on 8-well chamber slides (Millipore) for histology and 10^5^ cells were plated on 6-well plates (Costar Corning Incorporated) for molecular analysis. For histology, cells were fixed with 4% paraformaldehyde (Sigma-Aldrich Química S.A.) and stained with Alizarin Red after growing for 21 days in hMSC Ostegenic Differentiation Medium (Lonza) and 20%FBS/DMEM (as the control).

For three-dimensional differentiation, cell aggregates were formed by the hanging drop method, seeding drops containing 5 × 10^5^ cells on a lid of a 90 mm Petri dish filled with PBS (MP Biomedicals, Thermo Fisher Scientific). After two days, the aggregates were transferred to suspension culture system in propylene tubes (J.C. Catalán S.L., Barcelona, Spain) and maintained for 14 days in hMSC Chondrogenic Differentiation Medium (Lonza) with 10 ng/ml TFG-*β*3 (ProSpec-Tany TechnoGene) and then in hMSC Ostegenic Differentiation Medium (Lonza) for 21 days, or in 20%FBS/DMEM (as the control) for 35 days [48]. Aggregates were stained with Alizarin Red and Von Kossa to evaluate mineralization level and with Masson's Trichrome and Safranin O to investigate the presence of remaining chondrogenic features.

#### 2.7.2. Adipogenesis

For adipogenic cell differentiation experiments, 2 × 10^4^ cells were plated on 8-well chamber slides (Millipore) for histology and 10^5^ cells were plated on 6-well plates (Costar Corning Incorporated) for molecular analysis. Cells were grown for 21 days in hMSC Adipogenic Differentiation BulletKit Medium (Lonza) or StemPro Adipogenesis Differentiation Kit (Gibco, Thermo Fisher Scientific) and 20%FBS/DMEM (as the control). For histological analysis, cells were fixed with 4% paraformaldehyde (Sigma-Aldrich Química S.A.) before Oil Red O staining was performed. Slides were mounted with Glycergel aqueous mounting medium (Dako, Agilent Technologies Spain S.L.).

#### 2.7.3. Chondrogenesis

For chondrogenic cell differentiation experiments, three-dimensional cell culture was used [49]. Cell aggregates were formed by the hanging drop method and maintained for 21 days in hMSC Chondrogenic Differentiation Medium (Lonza) with 10 ng/ml human transforming growth factor *β*-3 (TFG-*β*3) (ProSpec-Tany TechnoGene, Rejovot, Israel) or in 20%FBS/DMEM (as the control). After that, aggregates were fixed with 4.0% formaldehyde (Panreac Química S.L.U., Barcelona, Spain), embedded in paraffin (Merck Millipore, Merck KGaA, Darmstadt, Germany), and cut in a microtome for histology. Extracellular matrix histological and immunohistochemical staining techniques were performed to assess the presence and nature of proteoglycans (Safranin O staining and aggrecan immunostaining) and collagen fibers (Masson's Trichrome staining and type II collagen immunostaining). Before immunostaining, cell aggregates were pretreated with chondroitinase ABC (Sigma-Aldrich Química S.A.). Incubations with primary antibodies anti-collagen II (clone 5B2.5 (1 : 25), Thermo Fisher Scientific) and anti-aggrecan (clone BC-3 (1 : 50), Abcam) were performed overnight. Dako REAL EnVision Detetion System (Dako, Agilent Technologies Spain S.L.) was used for immunostaining visualization, and slides were mounted with DPX mounting medium (Surgipath, Leica Microsistemas S.L., Spain).

#### 2.7.4. Histological and Immunohistological Assessment

All slides were observed employing an Olympus BX61 microscope (Olympus Iberia S.A.) coupled to an Olympus DP70 digital camera (Olympus Iberia S.A.). Micrographs were obtained employing the cellSens Dimension software (Olympus Iberia S.A.). Quantitative analysis of stained areas and intensity of staining was carried out as previously described [41] employing the ImageJ software (National Institutes of Health, Bethesda, USA). The percentage of stained area was expressed as mean ± standard error. Optical density (OD) was calculated as OD = log (max intensity/mean intensity), where log is the natural logarithm.

#### 2.7.5. Molecular Analysis

RNA from nondifferentiated cells as well as adipogenic and osteogenic cell differentiations was isolated by employing TRIzol Reagent (Thermo Fisher Scientific) and chloroform (Sigma-Aldrich Química S.A.) and precipitated with isopropanol (Sigma-Aldrich Química S.A.). Reverse transcription was carried out by using SuperScript VILO cDNA Synthesis kit (Thermo Fisher Scientific) in an Applied Biosystems Veriti 96-Well Thermal Cycler (Thermo Fisher Scientific), following the manufacturer's instructions. Quantitative real-time PCR (qPCR) was performed in a LightCycler1 480 Instrument (Roche), employing LightCycler 480 SYBR Green I Master (Roche) in addition to primers shown in Table 3. Tyrosine 3-monooxygenase/tryptophan 5-monooxygenase activation protein zeta (YWHAZ) was employed as a reference gene. Data analysis was done using the LightCycler 480 Relative Quantification software (Roche) and relative gene expression levels (REL) were calculated employing qbase+ software (Biogazelle, Zwijnaarde, Belgium). Normalized gene expression values were scaled to the sample with the highest expression for each gene. Osteogenesis- and adipogenesis-related gene expression levels are shown for each cell line as mean ± standard error, while transgene expressions are shown as mean ± standard error of their level of expression in four lines of transduced MSCs versus the four populations of primary MSCs from which they were derived.

### 2.8. Testing Colony Formation Ability and Oncogenic Potential of Transduced MSCs

To assess the colony formation ability of the transduced MSCs, cells were seeded in 6-well culture dishes (Costar Corning Incorporated) at a density of 500 cells per well and maintained in culture for one week. After that, the dishes were washed with PBS, fixed with 4% paraformaldehyde (Sigma-Aldrich Química S.A.) and stained with 0.1% crystal violet (Sigma-Aldrich Química S.A.). Micrographs were taken employing a Nikon SMZ 745 T stereomicroscope (Nikon Instruments Europe B.V., Amsterdam, Netherlands) coupled to a Nikon DS-Fi2 digital camera (Nikon Instruments Europe B.V.).

In addition, the soft agar colony formation assay [50] was performed to investigate the oncogenic potential of the transduced MSCs. For each cell line, 15000 cells were inoculated in 0.375% agar (Sigma-Aldrich Química S.A.) and layered on top of 0.5% agar layer in 12-well culture dishes (Costar Corning Incorporated) (3750 cells/well). Cells were incubated for 14 days at 37°C with 5% CO_2_. Colonies were observed and photographed using a Nikon Eclipse TS100 inverted microscope (Nikon Instruments Europe B.V.) coupled to a XM Full HD digital camera (Hangzhou Xiongmai Technologies (XM), Hangzhou, China).

Additionally, the level of expression of tumor-related genes was quantified in primary MSCs, transduced MSCs, and the osteosarcoma cell line 143B by quantitative real time PCR (qPCR). RNA isolation, qPCR, and data analysis were performed as previously described. Normalized gene expression values for each gene were scaled to the cell line 143B, employed as a positive control of oncogenic potential.

### 2.9. Statistical Analysis

Results were analysed using the Kruskal-Wallis and Mann–Whitney tests, employing Prism software (v5, GraphPad, Inc.). Differences between groups were considered significant if *p* value < 0.05.

## 3. Results

### 3.1. MSC Spinoculation with Retrovirus Produced by Phoenix Cells

Variations of several parameters for the transduction method were assayed for immortalization of primary human MSCs (Table 2). The spinoculation at 1000 ×*g* for 60 minutes with SV40LT and hTERT retrovirus (coinfection) produced by transfected Phoenix cells for 24 hours caused cell death of almost all cell population. Same result was obtained with hTERT retrovirus alone. A decrease in cell death was observed when employing SV40LT retrovirus alone, but even so spinoculation resulted in SV40LT-transduced MSCs (T-MSCs) with very low efficiency, and only with VPA induction of transgene expression (experiments 1 and 2).

Even when lowering centrifugation time to 45 minutes, spinoculation at 1000 ×*g* still produced cell death of most MSCs (experiment 3). Conversely, lowering centrifugation speed to 800 ×*g* resulted in much more cell survival after spinoculation (experiment 4). When prolonging incubation time for retrovirus production to 48 hours, the transduction efficiency was highly improved and a larger population of T-MSCs was obtained (experiment 4). A shorter time of centrifugation (30 minutes) reduced transduction efficiency and did not improve cell survival (experiment 5). Established parameters (48-hour incubation of transfected Phoenix cells for retrovirus production, spinoculation at 800 ×*g* for 45 minutes, and transgene expression induction by 2 mM VPA addition) were successfully employed for SV40LT transduction of MSCs derived from six donors (experiments 4, 6, and 8). Puromycin-selected T-MSCs had grown enough to be trypsinized within one week after selection.

The employment of these established parameters for a second transduction of T-MSCs with hTERT enabled us to obtain a small population of hTERT-transduced T-MSCs, which were named as immortalized MSCs (iMSCs). In our system, hTERT transduction is less efficient than SV40LT transduction, which is probably due to the fact that the hTERT plasmid is longer, and thus Phoenix cell transfection efficiency is reduced. It took 2-4 weeks for hygromycin-selected iMSCs to grow enough to be trypsinized and expanded but, after that, all the four generated iMSC lines (three OA and one non-OA lines, experiment 7) showed high proliferation rates.

### 3.2. Optimization of VPA Concentration

**Figure 2:**
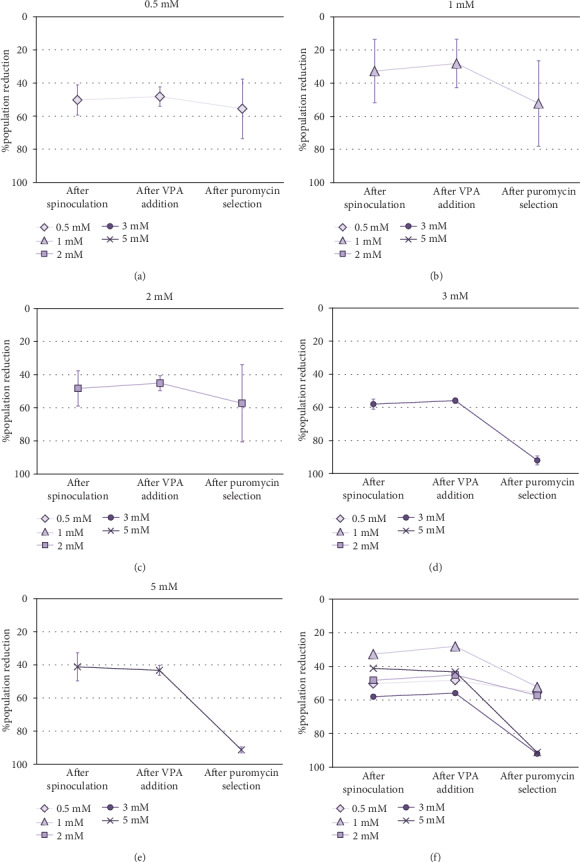
Percentage of cell population reduction (1) after spinoculation (*p* value = 0.1395), (2) after three-day incubation with 0.5 (a), 1 (b), 2 (c), 3 (d), or 5 (e) mM VPA (*p* value = 0.5615), and (3) after puromycin selection (*p* value = 0.0700) of SV40LT-transduced MSCs (f), inferred from percentage difference between absorbance measurements employing CCK-8 (measure wavelength: 450 nm; reference wavelength: 650 nm). The error bars represent the standard deviation of measurements for three different MSC cultures (*n* = 3).

Cell Counting Kit-8 (CCK-8) was employed to determine the optimal VPA concentration for transgene expression induction in MSCs. Taking into account that absorbance is proportional to cell population size, it was observed that the cell population was reduced after spinoculation in a very variable way (between 16% and 60%). This reduction was also observed in the cells that have been centrifuged without virus addition. After three-day incubation with different concentrations of VPA, little changes in cell population were observed (*p* value = 0.5615). After puromycin selection, a critical decrease of cell population size was noticed in all cases aside from 0.5 mM and 2 mM VPA previous treatments, in which this decrease was less severe. However, no significant differences were found between groups after treatment with different concentrations of VPA (*p* value = 0.7886) or after selection (*p* value = 0.0700). Absorbance measurements (1) before spinoculation, (2) after spinoculation, (3) after three-day incubation with 0.5-5 mM VPA, and (4) after puromycin selection, are shown in Supplementary Table S1. Differences between these measurements before and (1) after spinoculation, (2) after VPA treatment, and (3) after puromycin selection, are represented as the percentage reduction of population in Figure 2.

### 3.3. Testing SV40LT and eGFP-hTERT Nuclear Expression in Transduced MSCs

**Figure 3:**
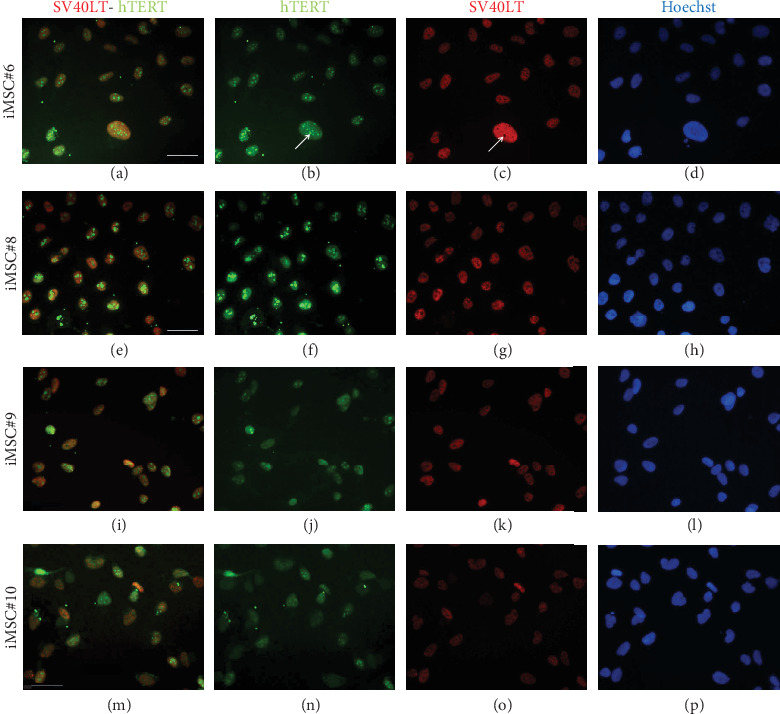
SV40LT and eGFP-hTERT immunostaining of iMSC#6 (a–d), iMSC#8 (e–h), iMSC#9 (i–l), and iMSC#10 (m–p). SV40LT is showed in red, eGFP-hTERT is showed in green, and Hoechst staining is showed in blue. Both nucleoli exclusion of SV40LT and nucleoli association of hTERT are pointed with white arrows. Scale bar: 50 *μ*m.

**Figure 4:**
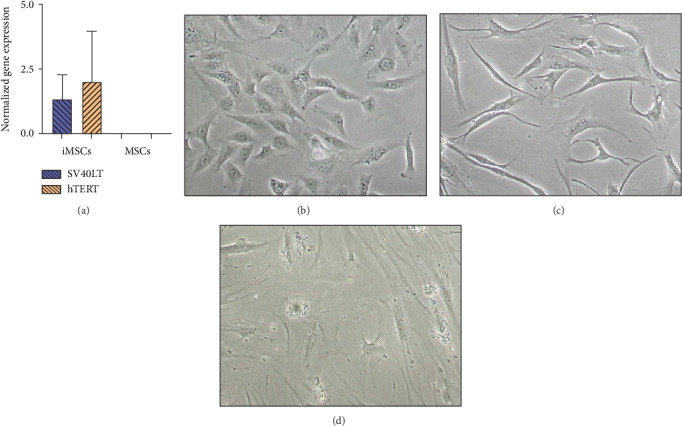
SV40LT and hTERT normalized expression in immortalized MSCs (iMSCs) and primary MSCs (a), shown as mean ± standard error for each group. Phase-contrast microscopic images of iMSCs (b), T-MSCs (c), and primary MSCs (d). Magnification 10x.

In order to prove that iMSCs were properly transduced and expressed both transgenes, SV40LT and GFP (fused to hTERT) immunostaining was performed. Expression of both transgenes was detected in the nuclei of iMSC#6, iMSC#8, iMSC#9, and iMSC#10. SV40LT exhibited a “nucleolar exclusion” expression pattern, while eGFP-hTERT showed a more variable pattern, with differences in intensity and location, including strong nucleolar signals and some more diffuse nucleoplasmic signals (Figure 3). Furthermore, SV40LT and hTERT expression was present in all the four iMSC lines and absent in the primary MSCs from which they were derived, as measured by qPCR. The REL of SV40LT in iMSC#6, iMSC#8, iMSC#9, and iMSC#10 were 2.713 ± 0.254, 0.986 ± 0.184, 1.000 ± 0.088, and 0.646 ± 0.061, respectively, and non-detected in primary MSCs, while the REL of hTERT were 2.676 ± 0.149, 4.392 ± 0.655, 1.000 ± 0.073, and 0.029 ± 0.002 in iMSC#6, iMSC#8, iMSC#9, and iMSC#10, respectively, 0.005 ± 0.001 in MSC#10 and non-detected in MSC#6, MSC#8, and MSC#9 (Figure 4(a)).

### 3.4. Analysis of Morphology and Proliferative Capacity of Transduced MSCs

**Figure 5:**
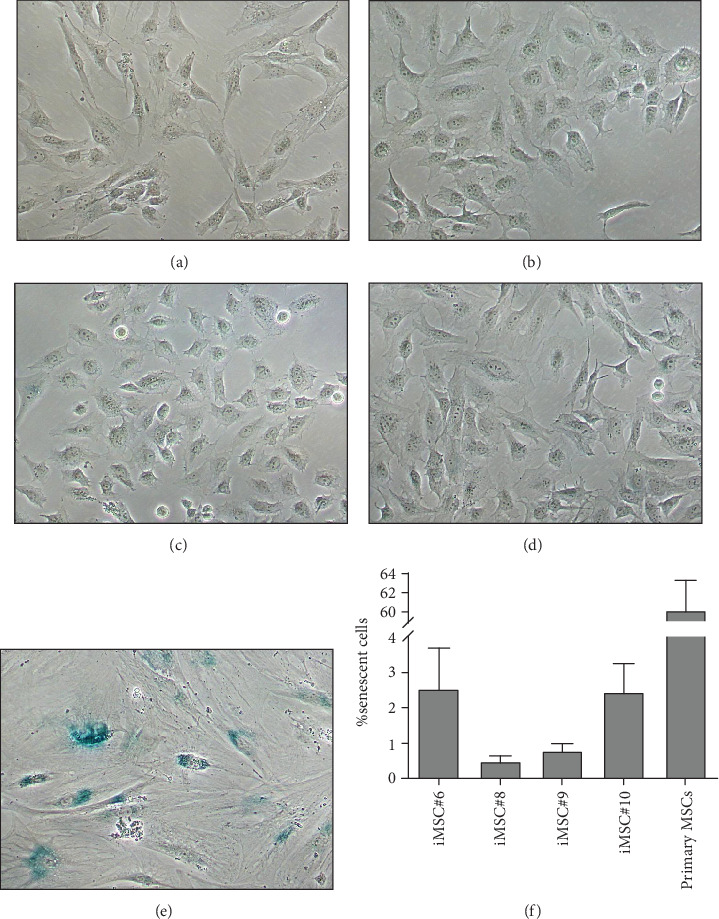
Phase-contrast microscopic images of the senescence-associated *ß*-galactosidase activity stained iMSC#6 (a), iMSC#8 (b), iMSC#9 (c), iMSC #10 (d) and primary MSCs (e). Magnification: 10x. Percentage of positive *ß*-galactosidase-stained, senescent cells, for each population (f). The error bars represent the standard deviation of measurements for three passages of each iMSC line (*n* = 3). Significant difference was found between iMSC lines and primary MSCs (*p* value < 0.0001).

Immortalized MSCs displayed a fibroblast-like cell morphology (Figure 4(b)) characteristic of MSCs. iMSCs showed more prominent nucleoli and less cytoplasm than T-MSCs (Figure 4(c)) and primary MSCs at the 4th passage (Figure 4(d)). OA and aged non-OA MSC cultures at the 4th passage presented 60% of cells acquired large and flat morphology (Figure 5(c)) and expressed senescence-associated *β*-galactosidase (Figures 5(e) and 5(f)), while iMSCs retained their size and fibroblast-like morphology and showed almost no *β*-galactosidase activity after more than 40 passages (Figures 5(a)–5(d)), being the percentage of *β*-galactosidase-positive cells 2.5 ± 1.1% for iMSC#6, 0.5 ± 0.1% for iMSC#8, 0.8 ± 0.2% for iMSC#9, and 2.4 ± 0.8% for iMSC#10 (Figure 5(f)). Regarding *β*-galactosidase activity, a significant difference was found between iMSC lines and primary MSCs (*p* value < 0.0001).

**Figure 6:**
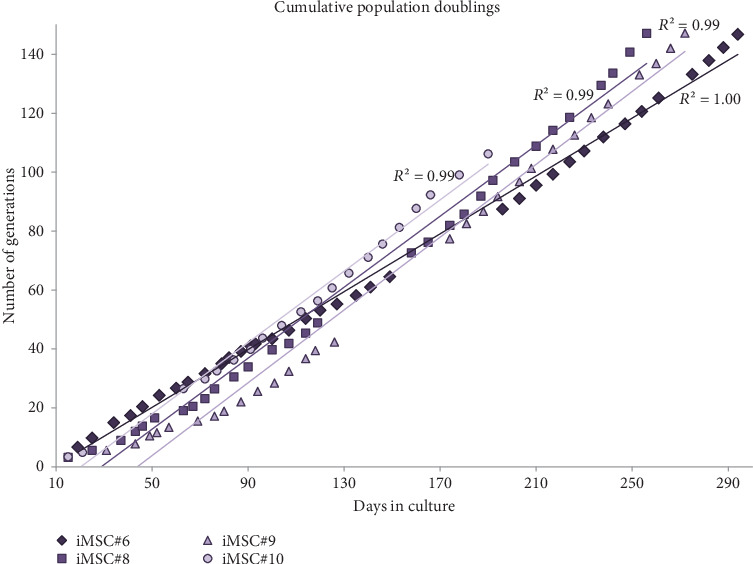
Number of generations accumulated by iMSC#6, iMSC#8, iMSC#9, and iMSC#10, calculated as (logNf–logNi)/log2 (where Nf is the final cell population, Ni is the number of cells in the inoculum, and log is the natural logarithm), facing days in culture. *R* > 0.99 and *p* value < 0.00005 for all four iMSC line growth curves.

The average of generation time for iMSCs was 2.0 days for iMSC#6 between passages 20 and 70, 1.9 days for iMSC#8 between passages 10 and 50, 2.3 days for iMSC#9 between passages 10 and 50, and 2.0 days for iMSC#10 between passages 10 and 35. In comparison, it was nearly 5.0 days in T-MSC#6 between passages 7 and 13 and almost 20.0 days in primary MSC#6 at the 4th passage. Immortalized MSCs #6, #8, #9, and #10 were grown over 100 generations for more than six months and continue to grow in culture. Regression analysis showed a constant proliferation rate, with coefficient of multiple correlation (*R*) > 0.99 and *p* value < 0.00005 for all four iMSC lines (Figure 6). Regarding generation time, no significant differences were found between iMSC lines (*p* value = 0.2).

### 3.5. Testing Maintenance of MSC Surface Marker Expression in Transduced MSCs

The expression of five MSC-positive (CD29, CD44, CD73, CD90, and CD105) and two negative (CD34 and CD45) surface markers was analysed in primary MSCs, T-MSCs, iMSCs, and in the 3a6 immortalized human MSC line. In all cases, >90% of the cells were positive for CD29, CD44, CD73, and CD90, except in T-MSC#8, T-MSC#9, and T-MSC#10 (86%, 70%, and 85% positive for CD73). Positivity for CD105 expression was more variable, reducing its expression from 85% to 74% in primary MSC#6 to iMSC#6 and from 92% to 38% in iMSC#8. Its expression in primary MSC#9 and iMSC#9 was 37-44% but 72% in T-MSC#9. In primary MSC#10, T-MSC#10, and iMSC#10, CD105 positivity was 76-74%, but it was raised to 82% in higher passage iMSC#10. T-MSCs, iMSCs, and 3a6 cells lacked CD34 expression while primary MSC#6 and MSC#8 were almost 10% CD34 positive. In all cases, <3% of the cells were positive for CD45 (Table 4).

### 3.6. Testing Maintenance of MSC Multipotency in Transduced MSCs

In order to investigate if transduced MSCs retained multipotency, primary MSC#6 (6th passage), T-MSC#6 (12th passage), iMSC#6 (20th and 65th passages), iMSC#8 (42th passage), and iMSC#9 (45th passage) were cultured under osteogenic and adipogenic differentiation conditions.

#### 3.6.1. Osteogenesis

**Figure 7:**
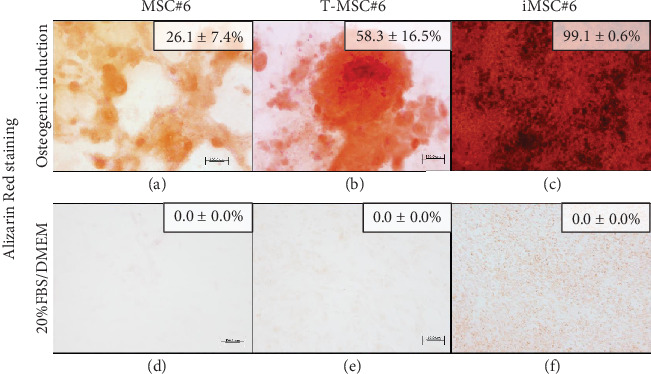
Alizarin Red staining of primary MSC#6, T-MSC#6, and iMSC#6 after 21 days of osteogenic induction (a–c) or culture in 20%FBS/DMEM (d–f). Percentage of Alizarin Red-stained area for each sample is shown. Scale bar: 100 *μ*m. Magnification: 10x.

**Figure 8:**
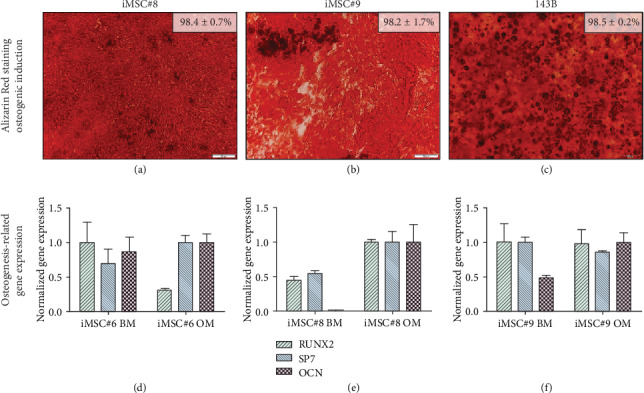
Alizarin Red staining of iMSC#8 (a), iMSC#9 (b), and 143B osteosarcoma cell line (c) after 21 days of osteogenic induction. Percentage of Alizarin Red-stained area for each sample is shown (scale bar: 100 *μ*m; magnification: 10x). Osteogenesis-related gene expression after culturing in basal medium (BM) and osteogenic medium (OM) is shown for iMSC#6 (d), iMSC#8 (e), and iMSC#9 (f) (*p* value > 0.05).

Primary MSC#6, T-MSC#6, and iMSCs were able to differentiate into the osteogenic lineage after 21 days of induction, as shown by calcium phosphate deposits stained in red with Alizarin Red. The lower mineralization area after osteogenic induction was detected in primary MSC#6 (Figure 7(a)). The mineralized area extension was twofold in T-MSC#6 (Figure 7(b)) and fourfold in iMSC#6 (Figure 7c()) compared to MSC#6. 143B osteosarcoma cell line (ATCC CRL-8303) was employed as a positive mineralization control for osteogenic differentiation experiments (Figure 8(c)). Furthermore, T-MSC#6 (11th passage), iMSC#6 (25th passage), iMSC#9 (50th passage), and iMSC#10 (40th passage) were cultured under chondrogenic differentiation conditions, and iMSC#6 (60th passage), iMSC#9 (50th passage), and iMSC#10 (40th passage) were also induced to differentiate towards the bone through endochondral ossification (Figure 7).

Mesenchymal cell lines iMSC#8 (Figure 8(a)) and iMSC#9 (Figure 8b) showed equal mineralization capacity to iMSC#6 and to 143B osteosarcoma cell line (Figure 8(c)). However, the osteogenic-related genes Runx2, Sp7 (Osterix), and Osteocalcin (OCN) were not upregulated in iMSC#6 cultured in osteogenic medium compared to basal medium (Figure 8(d)). Conversely, in iMSC#8, all the three genes tested were upregulated after osteogenic induction, especially the late osteogenic marker OCN (Figure 8(e)). In iMSC#9, only OCN was upregulated (Figure 8(f)). Due to this variability between iMSC lines, no significant differences in Runx2, Sp7, and OCN expression were found between osteogenically induced (OM) and control (BM) iMSCs (*p* value > 0.05). When comparing the expression in the three cell lines after osteogenic induction, iMSC#8 showed the highest expression of the three osteogenic markers.

**Figure 9:**
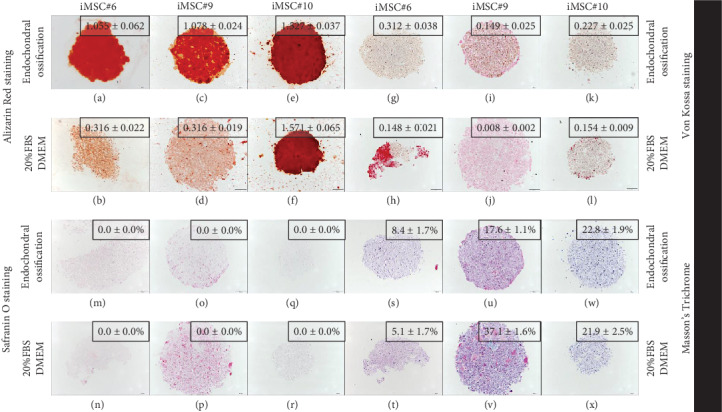
Alizarin Red staining (a–f), Von Kossa staining (g–l), Safranin O staining (m–r), and Masson's Trichrome staining (s–x) of iMSC#6, iMSC#9, and iMSC#10 after 3D osteogenic induction or five-week culturing in 20%FBS/DMEM) (scale bar: 100 *μ*m; magnification: 10x). Quantification is shown for each sample as percentage of stained area or intensity (optical density).

**Figure 10:**
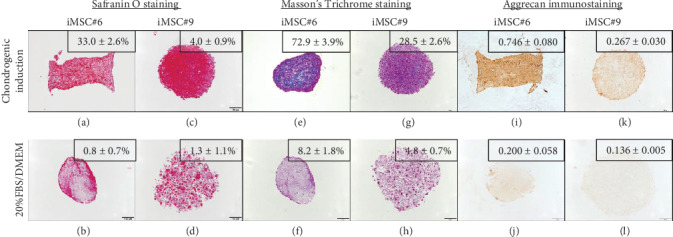
Histochemical and immunohistochemical staining of iMSC#6 and iMSC#9 after 21 days of chondrogenic induction or culturing in 20%FBS/DMEM: Safranin O staining (a–d), Masson's Trichrome staining (e–h), and aggrecan immunostaining (i–l) (scale bar: 100 *μ*m; magnification: 10x). Quantification is shown for each sample as percentage of stained area or intensity (optical density).

iMSC#6, iMSC#9, and iMSC#10 were also able to differentiate into the osteogenic lineage after induction in three-dimensional cell culture. iMSC#6 and iMSC#9 presented a threefold increase of Alizarin Red staining intensity (measured as OD) after differentiation in comparison with 20%FBS/DMEM control (Figures 9(a)–9(d)). In contrast, iMSC#10 presented the same intensity after osteogenic induction than culturing in 20%FBS/DMEM, as a result of the high mineralization of control sample (Figure 9(e) and 9(f)). All iMSCs presented higher Von Kossa staining intensities after osteogenic differentiation in comparison with respective controls (Figures 9(g)–9(l)). Proteoglycans were not detected in iMSC aggregates after neither osteogenic induction nor culturing in 20%FBS/DMEM for five weeks (Figures 9(m)–9(r)), in contrast to chondrogenic-induced iMSCs (Figures 10(a) and 10(c)). No significant increase or decrease of collagen fiber amount was detected after differentiation in comparison with five-week culturing in 20%FBS/DMEM (Figures 9(s)–9(x)).

#### 3.6.2. Adipogenesis

**Figure 11:**
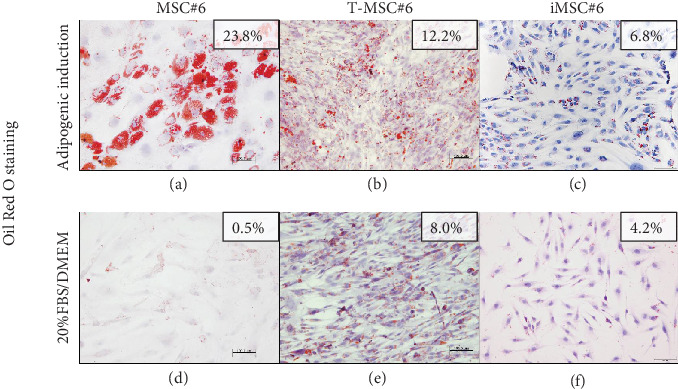
Oil Red O staining of primary MSC#6, T-MSC#6, and iMSC#6 after 21 days of adipogenic induction (a–c) or culture in 20%FBS/DMEM (d–f). Percentage of Oil Red O-stained area for each sample is shown. Scale bar: 100 *μ*m. Magnification: 10x.

Primary MSC#6 showed the highest potential to differentiate into the adipogenic lineage after 21 days of induction, as shown by intracellular lipid droplets stained in red with Oil Red O (Figure 11(a)). T-MSC#6 showed a 50% reduction of the stained area in comparison with primary MSC#6, and few preadipocytes could be identified (Figure 11(b)). iMSC#6 retained the adipogenic differentiation potential, but the preadipocytes formed, though clearly identifiable, were less mature than those formed by primary MSC#6 and contained smaller lipid vacuoles (Figure 11(c)). Accordingly, iMSC#6 showed a 70% reduction of the stained area in comparison with primary MSC#6. Of note, positive Oil Red O staining was observed in T-MSC#6 (Figure 11(e)) and also in iMSC#6 (Figure 11(f)) after culturing in 20%FBS/DMEM, suggesting spontaneous adipogenic differentiation.

**Figure 12:**
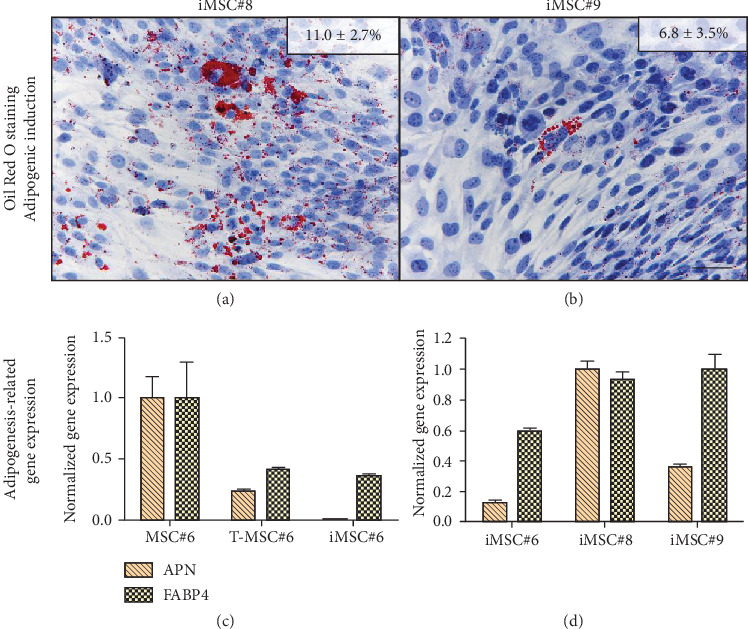
Oil Red O staining of iMSC#8 (a) and iMSC#9 (b). Percentage of Oil Red O-stained area for each sample is shown (scale bar: 100 *μ*m; magnification: 10x). Adipogenesis-related gene expression after culturing in adipogenic medium is shown comparing MSC#6, T-MSC#6, and iMSC#6 (c) and comparing iMSC#6, iMSC#8, and iMSC#9 (d). APN and FABP4 expression was not detected in cells cultured in basal medium.

Cell line iMSC#8 showed a percentage of Oil Red O-stained area similar to T-MSC#6 (Figure 12(a)), while iMSC#9-stained area was equal to iMSC#6 (Figure 12(b)). The reduction of the adipogenic potential from primary MSC#6 to iMSC#6 was supported by gene expression analysis, which showed reduced expression of the adipogenic markers adiponectin (APN) and FABP4 (Figure 12(c)). When comparing the expression of the three cell lines after adipogenic induction, iMSC#6 showed the lowest expression of both adipogenic markers, and iMSC#8 showed the highest adipogenic potency (Figure 12(d)). Expression of APN and FABP4 was not detected in cells cultured in basal medium (BM).

#### 3.6.3. Chondrogenesis

**Figure 13:**
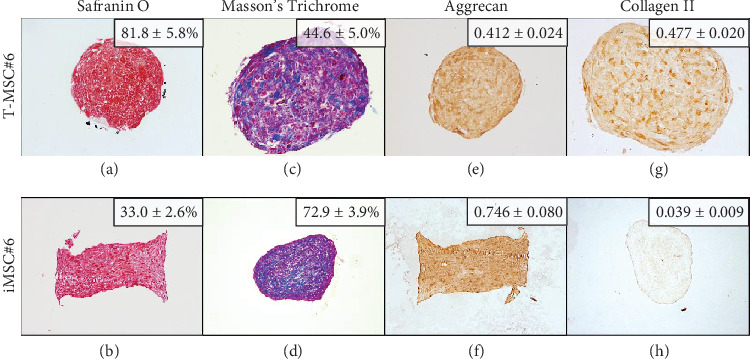
Histochemical and immunohistochemical staining of T-MSC#6 and iMSC#6 aggregates after 21 days of chondrogenic induction: Safranin O staining of T-MSC#6 (a, 20x) and iMSC#6 (b, 10x); Masson's Trichrome staining of T-MSC#6 (c, 20x) and iMSC#6 (d, 10x); aggrecan immunostaining of T-MSC#6 (e, 20x) and iMSC#6 (f, 10x); and type II collagen immunostaining of T-MSC#6 (g, 20x) and iMSC#6 (h, 10x). Quantification is shown for each sample as percentage of stained area or intensity (optical density).

Both T-MSC#6 and iMSC#6 were able to differentiate into the chondrogenic lineage after 21 days of induction in three-dimensional cell culture. Histological staining indicated the presence of proteoglycans (stained orange by Safranin O staining; Figures 13(a) and 13(b)) and collagen (stained blue by Masson's Trichrome staining; Figures 13(c) and 13(d)) in the extracellular matrix of the aggregates. Strong aggrecan immunostaining was observed in both T-MSC#6 (Figure 13(e)) and iMSC#6 (Figure 13(f)) aggregates, being almost twofold more intense in iMSC#6. Type II collagen immunostaining was intense in T-MSC#6 aggregates (Figure 13(g)) but faint in iMSC#6 (Figure 13(h)), immunostaining being twelvefold more intense in T-MSC#6 aggregates. Aggregates had heterogeneous shapes and sizes, as can be appreciated in the micrographs (Figure 13).

When compared to respective controls, both iMSC#6 and iMSC#9 aggregates contained more proteoglycans when chondrogenic differentiation was induced, as shown by orange staining of proteoglycans by Safranin O staining (Figures 10(a)–10(d)). Greater amount of collagen was also detected in chondrogenic-induced iMSCs, as shown by blue staining of collagen fibers by Masson's Trichrome staining (Figures 10(e)–10(h)). Aggrecan immunostaining was almost fourfold and twofold more intense in chondrogenic-induced iMSC#6 and iMSC#9, respectively, when compared to the controls (Figures 10(i)–10(l)). When comparing iMSC#6 and iMSC#9, iMSC#6 showed the most intense aggrecan immunostaining after chondrogenic induction, correlating with the highest amount of proteoglycans detected. Chondrogenic-induced iMSC#10 aggregates were too small to allow the performance of histological techniques, and therefore could not be analysed. iMSC#8 was unable to form aggregates by the hanging drop method employed.

### 3.7. Testing Colony Formation Ability and Oncogenic Potential of Transduced MSCs

**Figure 14:**
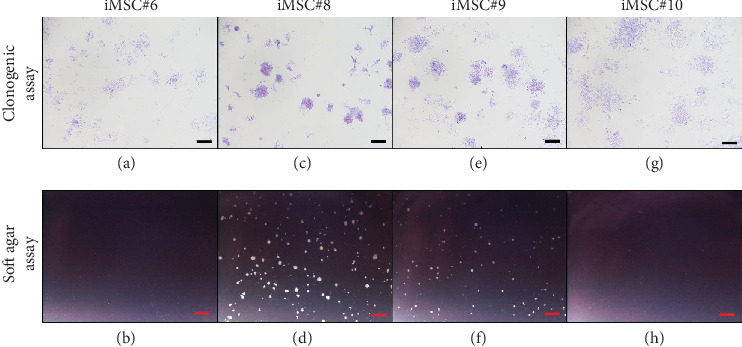
Crystal violet staining to assess the clonogenic potential of iMSC lines (a, c, e, g) and soft agar assay to assess their oncogenic potential (b, d, f, h). Scale bar: 1 mm.

A clonogenic assay was performed to assess the colony formation ability of iMSCs. After one week, all iMSC lines were able to form colonies. In addition, a soft agar assay was performed to assess oncogenic potential of iMSCs. After 14 days, two out of four iMSC lines formed colonies in soft agar. Representative micrographs are shown in Figure 14. iMSC#6 formed colonies with wide intercellular spaces (Figure 14(a)) and was not able to grow in soft agar (Figure 14(b)). Conversely, iMSC#8 formed more compact colonies (Figure 14(c)) and was able to grow in soft agar (Figure 14(d)), as well as iMSC#9 (Figures 14(e) and 14(f)). iMSC#10 presented a phenotype similar to iMSC#6, forming uncompacted colonies (Figure 14(g)) and being unable to grow in soft agar (Figure 14(h)).

As for tumor-related gene expression, no significant differences were found between iMSCs and primary MSCs (*p* value > 0.05). However, some trends were observed. The REL of the tumor suppressor p53 was upregulated in three out of four iMSC lines, iMSC#6 (0.599 ± 0.049), iMSC#9 (0.599 ± 0.023), and iMSC#10 (0.766 ± 0.163) in comparison with the REL of their respective untransduced counterparts (0.017 ± 0.001, 0.286 ± 0.034, and 0.268 ± 0.036, respectively), with a REL lower than that of 143B (1.000 ± 0.059), while in iMSC#8 it was downregulated (0.310 ± 0.076 versus 0.963 ± 0.054 in MSC#8). Similarly, the REL of the tumor suppressor Rb was higher in three of the iMSC lines, iMSC#6 (1.424 ± 0.064 versus 0.187 ± 0.006 in MSC#6), iMSC#8 (2.540 ± 0.407 versus 1.682 ± 0.260 in MSC#8), and iMSC#10 (2.412 ± 0.452 versus 0.874 ± 0.004 in MSC#10), but lower in iMSC#9 (1.371 ± 0.113 versus 1.892 ± 0.118 in MSC#9).

**Figure 15:**
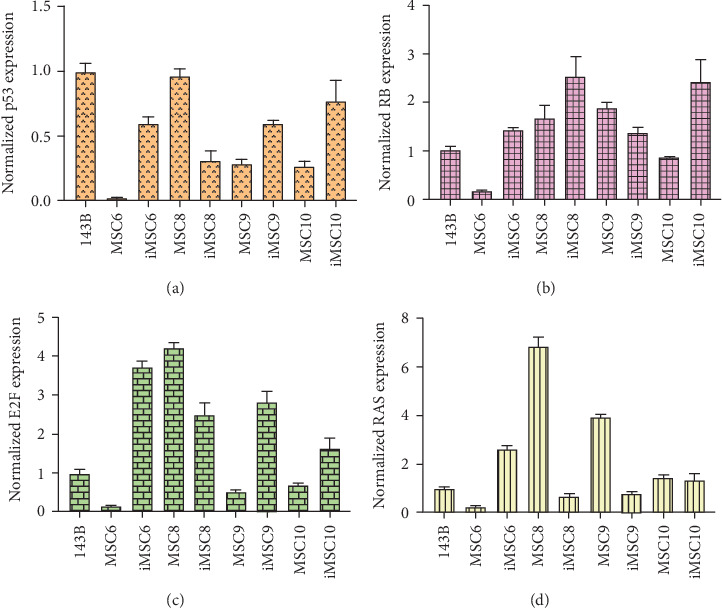
Normalized levels of expression of the tumor-related genes p53, Rb, E2F1, and H-RAS in primary MSCs, immortalized MSCs (iMSCs), and the osteosarcoma cell line 143B (*p* value > 0.05).

The REL of the transcription factor E2F1, a positive regulator of cell proliferation, was also higher in three of the iMSC lines, iMSC#6 (3.745 ± 0.130 versus 0.151 ± 0.014 in MSC#6), iMSC#9 (2.819 ± 0.270 versus 0.525 ± 0.028 in MSC#9), and iMSC#10 (1.653 ± 0.239 versus 0.693 ± 0.051 in MSC#10), and lower in iMSC#8 (2.505 ± 0.304 versus 4.213 ± 0.138). Contrariwise, the protooncogene H-RAS was only upregulated in iMSC#6 (REL 2.612 ± 0.143 versus 0.255 ± 0.025 in MSC#6) but downregulated in iMSC#8 (0.662 ± 0.140 versus 6.845 ± 0.383 in MSC#8) and iMSC#9 (0.785 ± 0.083 versus 3.959 ± 0.082 in MSC#9) and maintained in iMSC#10 (1.352 ± 0.247 versus 1.464 ± 0.102 in MSC#10) (Figure 15).

## 4. Discussion

MSCs derived from aged donors are prone to senesce during *in vitro* culture but could acquire an unlimited proliferation potential if both p53- and Rb-mediated pathways and telomere shortening are repressed by transduction of immortalization genes [3, 15, 18]. Retroviral transduction of slow-dividing adult human cells is an ineffective process and requires the use of enhancing methods such as spinoculation, employment of chemical adjuvants, and addition of transgene expression inductors, which give better results when adapted to the particular transduction system [20, 37]. The method here proposed is suitable for transduction of aged MSCs and other types of adult human cells and allows their immortalization. For its optimization, several variations were assayed, including SV40LT and hTERT cotransduction and sequential transduction, two incubation times of packaging cells for retrovirus production, two speeds and three time-points of centrifugation for spinoculation, and five different concentrations of VPA, an inductor of transgene expression (Table 2).

In our system, spinoculation of primary MSCs with hTERT retrovirus triggered general cell death, which may be due to apoptosis induction by hTERT overexpression, a phenomenon that has been observed in primary cells with a short lifespan [16]. Even when employing SV40LT retrovirus, cell survival to spinoculation at 1000 ×*g* was low, which may be attributed to centrifugation-induced stress, as lowering centrifugation speed to 800 ×*g* remarkably improved cell survival. The maximum optimal spinoculation speed depends on cell type [23]; at higher speeds, centrifugation-induced stress might cause damage to the cells and make them more susceptible to HDMB toxicity [35]. However, lowering centrifugation time below 45 minutes did not improve cell survival and was unfavourable for infection, in accordance with the results obtained by others authors [33, 34].

Six lines of SV40LT-transduced MSCs (T-MSCs) were generated employing the chosen parameters for spinoculation (800 ×*g* and 45 minutes) and SV40LT retrovirus produced by Phoenix cells during 48-hour incubation at 32°C. Employing hTERT retrovirus and these same parameters for T-MSC transduction, hTERT-induced apoptosis was not observed, which is in accordance with previous reports [16]. In this manner, four different lines of immortalized MSCs (iMSCs) were obtained.

Because of their polyanionic nature, viral DNA interacts with positively charged histones in the nucleus, resulting in a loss of gene expression, which may be a cellular defence mechanism against viruses. However, these gene silencing can be reversed by histone acetylation through the addition of histone deacetylase inhibitors such as VPA [51, 52]. Cell viability measurements performed to optimize VPA concentration showed that MSC population was reduced after spinoculation in a very variable way. This reduction may be attributed to centrifugation instead of infection, since it was also observed when no viruses were added. Our results indicated that VPA do not exert any negative effect on cell viability, but a negative effect on proliferation while present in the culture medium cannot be discarded.

After puromycin selection of T-MSCs, cell population size decreased for all VPA concentrations tested. However, MSCs treated with 0.5 mM and 2 mM VPA showed the most moderate reduction of cell population. We hypothesized that it can be related to differences in spinoculation efficiency caused by the particular position of each well of the dish when centrifuging, which, to the best of our knowledge, have never been reported. It is also important to note that during transduction, transgenes are being inserted in different regions of the genome, with different levels of epigenetic modifications, in each transduced cell. Therefore, each cell may respond differently to the same concentration of VPA [53], probably contributing to the variability observed.

Finally, 2 mM VPA was established as the optimal concentration above 0.5 mM based on our previous observations and its greater similarity to concentrations established as optimal for other transduction systems [37, 40]. For example, Cervera et al. [37] reported that the addition of 3.36 mM VPA four hours posttransfection was the optimal concentration to enhance transient gene expression while increasing cell viability in HEK-293 cells [37], and Fang et al. [38] employed a concentration of 3.5 mM VPA to increase the production of recombinant antibodies with similar results [38]. In addition, Joglekar et al. [51] showed that VPA enhanced gene expression from lentiviral vectors in human hematopoietic stem cells in a concentration-dependent manner, with 1.5 mM being significantly superior to 0.5 mM [51].

However, Jäger et al. [40] observed a reduction of cell proliferation and viability along with the increase in recombinant antibody production in HEK-293 cells after treating with 3.75 mM VPA [40], and Wulhfard et al. [39] also observed that 3.75 or 5 mM VPA induced a concentration-dependent cell growth arrest in CHO cells, despite increasing recombinant antibody yields [39]. All these data indicated that relatively high VPA concentrations (between 1.5 and 3.5 mM) may enhance transgene expression more efficiently, but at higher concentrations (over 3.5 mM), the deleterious effects over cells overcome the beneficial effects over transgene expression.

Immunofluorescence of iMSC#6, iMSC#8, iMSC#9, and iMSC #10 showed that, in their nuclei, SV40LT was located in the nucleoplasm and excluded from the nucleoli, which occurs in other SV40LT-transduced cell lines [45]. Catalytically active telomerase intranuclear localization is regulated by cell cycle stage, being sequestered at nucleoli in the G1 phase and released to the nucleoplasm in the S/G2 phase. Although it has been reported that SV40LT transduction of primary cells promotes the release of telomerase from the nucleoli to the nucleoplasm, in these iMSCs, eGFP-hTERT seems to be preferentially associated with the nucleoli (Figure 3), suggesting that they have not underwent malignant transformation [45] despite immortalization.

However, iMSC#8 and iMSC#9 were able to form colonies in soft agar in spite of the H-RAS downregulation in both cell lines after immortalization. This ability may be related to oncogenic alterations present in the primary MSCs #8 and #9, which expressed levels of H-RAS much higher than the osteosarcoma cell line 143B. Nevertheless, immortalized MSCs transduced with protooncogenes can eventually become tumorigenic, making them useless for clinical approaches, but not for research purposes. Oncogenic mutations may arise during passaging of iMSCs [54], and iMSCs seeded at low densities during long periods of time have been reported to be tumorigenic [55].

Growth arrest is orchestrated by the tumor suppressors p53 and Rb. In response to stress or DNA damage, p53 is phosphorylated and liberated from its binding to E3 ubiquitin ligase Mdm2, hence activating the senescence pathways. During quiescence, unphosphorylated Rb proteins control cell proliferation by binding and inhibiting E2F transcription factors, therefore blocking cell cycle progression. SV40LT binds to both these proteins, allowing the overcoming of senescence and releasing the activity of E2F transcription factors [54, 56]. Since SV40LT inhibition of p53 and Rb occurs at the protein level, the mRNA coding for these proteins may accumulate in SV40LT-transduced cells without effectively triggering senescence. In fact, higher levels of p53 and Rb in iMSCs do not correlate with lower levels of E2F nor have any effect over cell proliferation. Other tumor suppressor genes, such as PTEN, have also been found to be upregulated after MSC immortalization [57].

We found that MSC morphology did not change after transduction of immortalization genes, as has been previously reported by others [9, 14, 19]. As for their morphology, iMSCs were more similar to young MSCs than aged MSCs, retained the ability to grow until reaching a confluent monolayer, and lacked granular content related to lisosomal senescence-associated *β*-galactosidase activity (≤5.0% of cells were *β*-galactosidase positive), unlike late-passage primary OA MSCs, as has also been observed in other immortalized MSC lines [14, 17].

All the four iMSC lines showed a higher proliferation rate than T-MSC#6 (~2 and 5 days, respectively), which showed likewise a higher proliferation rate than primary MSC#6. Faster proliferation has been often observed after SV40LT transduction [9] but has not been attributed to hTERT alone [14], yet the combination of SV40LT or p53 knockdown with hTERT overexpression can exert a synergistic effect in improving MSC growth rate [17, 19]. From the initial passage to senescence, MSCs are not able to carry out more than 30-40 [5, 12, 14], while immortalized MSCs are able to reach more than 200 PDs [12, 14]. Up to now, all the four iMSC lines have undergone more than 100 PDs and continue to proliferate *in vitro* without any sign of senescence.

As for phenotypic characterization, the mesenchymal surface markers CD29, CD44, CD73, and CD90 were highly expressed in primary MSCs, T-MSCs, and iMSCs, while CD105 expression was reduced with either subculturing or transduction in iMSC#6 and iMSC#8, and also showed varying levels of expression iMSC#9. However, these markers are also expressed by terminally differentiated MSCs [58] and other cells such as fibroblasts [2, 59], and thus, they may not be very reliable markers to identify undifferentiated MSCs, although traditionally established as a necessary requirement for defining them [60].

Even though it has been generally reported that the high expression of CD29, CD44, CD73, CD90, and CD105 is maintained in MSCs regardless of passage number [5, 18], some authors have noticed the downregulation of some of these markers during *in vitro* expansion [61, 62] and due to particular culture conditions [63]. Their expression seems to be mainly preserved after immortalization [12, 14, 19], although CD105 decrease following SV40LT and hTERT transduction has also been reported [17] and has also been observed in the E6/E7 and hTERT-transduced 3a6 cell line. Of notice, CD105 upregulation has been associated with late passages and cellular senescence [64]. The reason of its decrease in iMSCs is difficult to elucidate as *in vitro* culture-related and immortalization-related effects are undistinguishable.

Immortalized MSCs have often been reported to be able to differentiate towards osteoblasts and adipocytes [12, 9, 14, 19], but transduction of SV40LT and hTERT together has resulted in immortalized MSCs without adipogenic [18] or osteogenic [17] potential. The adipogenic potential has been described to be progressively reduced throughout *in vitro* expansions in spite of immortalization [18, 65]. Chondrogenic potential of immortalized MSCs has seldom been assayed [9, 17, 18], and when investigated, has often shown to be limited [12, 14, 66, 67], with scarce exceptions [19].

iMSC#6 differentiation potential seems to have varied as a result of either immortalization or passaging. Osteogenic potential was higher in T-MSC#6 than in primary MSC#6, and even greater in iMSC#6. On the contrary, T-MSC#6 and iMSC#6 showed reduced adipogenic potential in comparison with MSC#6. Osteogenic and adipogenic potential of iMSC#8 and iMSC#9 were similar to that of iMSC#6, except for the higher adipogenic potential of iMSC#8. Chondrogenic potential of T-MSC#6, iMSC#6, and iMSC#9 was evidenced by their ability to produce an extracellular matrix containing collagen and aggrecan. However, notably higher amount of type II collagen was detected in T-MSC#6 aggregates, perhaps indicating that iMSCs produced a lower quality cartilage-like tissue.

The lower quality of cartilage-like tissue produced by iMSC#6 and iMSC#9 in comparison with T-MSC#6 could be suggested as a result of its lower CD105 positivity, as it is part of the TGF-*ß* receptor complex. However, it has been noted that CD105^+^ MSCs have no superior chondrogenic potential than the CD105^−^ ones [68], even though it is still controversial [69]. It has also been stated that, as a counterpart of decreased chondrogenic potential, CD105^−^ MSCs show increased osteogenic potential [69] as a result of superior adhesion and mineralization efficiency [70], which might be related to the superior osteogenic potential of iMSCs. Nevertheless, surface marker expression does not necessarily reflect the functional capacities of MSC [63], and the inferior osteogenic potential of primary MSC#6 may be also due to them being differentiated at the 6th passage, when cells are expected to be aged [5, 71].

The differentiation abilities of T-MSCs and iMSCs may be derived from the potential of those MSCs effectively transduced when spinoculations were performed [12], since those cells, susceptible to infection and located on the parts of the dish where less centrifugation-induced stress is exerted, are arbitrary selected during spinoculation. Moreover, extensive passaging may lead to the selection of those cells with higher growth rates, altering thereby other properties of the cell population, as multilineage differentiation potential [62]. It is commonly accepted that osteogenesis is the default differentiation pathway for MSCs [12, 72, 73] and the most commonly retained differentiation lineage at later passages [62, 72]. This osteogenic commitment may be responsible of the high bone-related transcription factor expression detected before osteogenic inducement of iMSCs.

In summary, we developed a method that combines enhancement of retroviral transduction by spinoculation and induction of transgene expression by VPA supplementation. This method allows immortalization of aged MSCs with preservation of most mesenchymal features. However, it has been noticed that either immortalization or passaging produced some alterations regarding CD105 expression and multidifferentiation potential.

## 5. Conclusions

The aim of this study was to develop an easy and efficient method for immortalization of slow-dividing cells, such as aged MSCs. MSCs derived from aged and OA donors may be immortalized by SV40LT and hTERT sequential transduction employing the method we described. Immortalization allows these cells to increase its proliferation rate and to avoid senescence, while maintaining mesenchymal phenotype and multipotency.

The immortalized MSC lines generated by this method have the ability to differentiate into the osteogenic lineage both in two-dimensional and three-dimensional cultures, and also into the adipogenic and chondrogenic lineages with several levels of success. Therefore, iMSCs are expected to be valuable tools for the bone and cartilage regeneration research. Specifically, further analysis can shed light about pathological differences in the field of rheumatic diseases, as the cells can be derived from the MSCs of aged and OA donors.

## Figures and Tables

**Table 1:** List of spinoculation experiments found in the literature, detailing spinoculation conditions, chemical adjuvants employed, target cell type, type of virus used, and packaging cells employed to produce them. HDMB: hexadimethrine bromide; PEG: polyethylene glycol; RT: room temperature.

Target cell type	Packaging cells	Virus	Adjuvants	Spinoculation conditions	Objective	Reference
Peripheral blood mononuclear cells (PBMCs)	293 T cells	Lentivirus	10 *μ*g/ml HDMB and 1000 *μ*g/ml P338	800 ×*g* 90 min RT	Optimizing transduction of haematopoietic cells *in vitro*	[22]
CD3^+^ T cells	293 T cells	Lentivirus	8 *μ*g/ml HDMB	800 ×*g* 90 min 32°C	Transducing human T cells	[28]
T follicular helper cells	293 T cells	Human immunodeficiency virus (HIV)	None	1200 ×*g* 120 min RT	Investigating T follicular helper cells permissivity to HIV *ex vivo*	[26]
Hematopoietic cells, lymphoid cell lines, and thymocytes	293 T cells	Retrovirus or lentivirus	5 *μ*g/ml HDMB	460 ×*g* 60-90 min RT	Presenting protocols to transduce lymphoid progenitors with viral vectors	[20]
Peripheral blood mononuclear cells (PBMCs)	293 T cells	HIV	None	1200 ×*g* 120 min 30 °C	Determining whether medroxyprogesterone acetate increases HIV infection of unstimulated PBMCs	[30]
HBV receptor-complemented HepG2 cell line	HepDE19 cells	Hepatitis B virus (HBV)	4% PEG-8000	1000 ×*g* 60 min RT	Establishing an *in vitro* model of HBV infection	[33]
Lamina propria mononuclear cells (LPMCs)	MOLT4-CCR5 cells	HIV	None	1200 ×*g* 120 min RT	Modelling the interactions of gut LPMCs, HIV-1, and *Escherichia coli*	[32]
HIV Rev-dependent GFP indicator cell line (Rev-CEM) and primary CD4 T cells	HeLa cells	HIV	None	300-1200 ×*g* 2 hours RT, with Rev-CEM cells showing greater enhancement at 600 ×*g* and primary CD4 T cells showing greater enhancement at 300 ×*g*	Examining the response of cortical actin to centrifugal pressure and ensuing effects on HIV-1 infection	[23]
Keratinocytes and fibroblasts	Phoenix cells	Retrovirus	1-5 *μ*g/ml HDMB	Two spinoculations at 750 ×*g* 45 min 24 hours apart	Generating induced pluripotent stem cells	[29]; [21]
Hepatic cell line Huh7.5.1	Huh7 cells	Hepatitis C virus (HCV)	None	500-1000 ×*g* 30-120 min RT, showing greater enhancement of infection at 1000 ×*g* 120 min	Investigating the effect of low-speed centrifugation on HCV infection of human hepatocytes	[34]
Transfected CHO-K1 and CHO-745 cells	Vero cells	Herpes simplex virus type-1 (HSV-1)	None	1200 ×*g* 120 min 37°C	Examining the dependence of HSV-1 entry receptors on heparan sulfate	[31]
Haematopoietic cells	Phoenix cells	Retrovirus	0.8 *μ*g/ml HDMB	Two spinoculations of 45 min RT 6 hours apart; repeated 1-4 days depending on cell type	Characterizing the efficiency of gene transfer in human haemopoietic cells using the Pinco-Phoenix system	[25]
Feline whole fetus cells (fcwf-4)	—	Feline infectious peritonitis virus (FIPV) grown in fcwf-4	None	400 ×*g* 0-180 min RT, with increasing enhancement until 120 min of spinoculation	Investigating the effect of centrifugation on the ability of FIPV to infect cells in culture	[24]
NIH-3T3 TK^−^ and HUT 78 cells (human T cell leukemia line)	22 producer cell lines derived from PA317 line	Retrovirus	8 *μ*g/ml HDMB	1600 ×*g* 90 min 32°C	Optimizing methods of retroviral vector production and transduction	[27]

**Table 2:** Variations of the transduction method assayed, detailing retrovirus employed, culture conditions for Phoenix cells, spinoculation conditions, and VPA concentration employed to induce transgene expression.

Experiment	Cells	Retrovirus	*φ*NX-A culture	Spinoculation	VPA (mM)
1	MSC#1 MSC#2	SV40LT+hTERT	32°C 24 h	1000 ×*g* 60 min	0
2	MSC#1 MSC#3	(1) SV40LT+hTERT (2) SV40LT (3) hTERT	32°C 24 h	1000 ×*g* 60 min	0-0.5
3	MSC#4 MSC#5	SV40LT	32°C 24 h	1000 ×*g* 45 min	0.5-2
4	MSC#6	SV40LT	32°C 48 h	800 ×*g* 45 min	0.5-2
5	MSC#7 MSC#8	SV40LT	32°C 48 h	800 ×*g* 30 min	1-3
6	MSC#9 MSC#1 MSC#4 MSC#10	SV40LT	32°C 48 h	800 ×*g* 45 min	2
7	T-MSC#6 T-MSC#8 T-MSC#9 T-MSC#10	hTERT	32°C 48 h	800 ×*g* 45 min	2
8	MSC#11 MSC#12 MSC#13	SV40LT	32°C 48 h	800 ×*g* 45 min	0.5-5

**Table 3:** List of primers employed for quantitative real time PCR (qPCR) analysis.

Gene	Reference sequence	Forward primer (5′ ⟶ 3′)	Reverse primer (5′ ⟶ 3′)
Tyrosine 3-monooxygenase/tryptophan 5-monooxygenase activation protein zeta (YWHAZ)	NM_003406.3	GATCCCCAATGCTTCACAAG	TGCTTGTTGTGACTGATCGAC
Homo sapiens runt-related transcription factor 2 (RUNX2)	NM_001024630.4	TTACTTACACCCCGCCAGTC	TATGGAGTGCTGCTGGTCTG
Homo sapiens Sp7 transcription factor (SP7)	NM_001173467.2	TCCCCTGTTGCCATGGTTAT	CCACCCATTCTTCAGGAGGT
Homo sapiens bone gamma-carboxyglutamate protein (OCN)	NM_199173.5	GGCGCTACCTGTATCAATGG	TCAGCCAACTCGTCACAGTC
Homo sapiens adiponectin, C1Q and collagen domain containing (APN)	NM_001177800.1	GGTGAGAAAGGAGATCCAGGT	TGCTGAGCGGTATACATAGGC
Homo sapiens fatty acid-binding protein 4 (FABP4)	NM_001442.2	GGATGATAAACTGGTGGTGGA	CACAGAATGTTGTAGAGTTCAATGC
Homo sapiens HRas protooncogene, GTPase (HRAS)	NM_005343.4	TGCCATCAACAACACCAAGT	ACGTCATCCGAGTCCTTCAC
Homo sapiens tumor protein p53 (p53)	NM_000546.5	GGCCCACTTCACCGTACTAA	GTGGTTTCAAGGCCAGATGT
Homo sapiens RB transcriptional corepressor 1 (RB1)	NM_000321.2	TGCATGGCTCTCAGATTCAC	AGTTGGTCCTTCTCGGTCCT
Homo sapiens E2F transcription factor 1 (E2F1)	NM_005225.3	TGTGCATGAGTCCATGTGTG	GGCCGAAAGTGCAGTTAGAG
Simian virus 40 (SV40)	NC_001669.1	TGGGGAGAAGAACATGGAAG	AAATGAGCCTTGGGACTGTG
Homo sapiens telomerase reverse transcriptase (hTERT)	NM_198253.3	GCTAGTGGACCCCGAAGG	CCTCCCTGACGCTATGGTT

**Table 4:** Flow cytometric analysis of mesenchymal markers CD29, CD44, CD73, CD90, and CD105 and the hematopoietic markers CD34 and CD45; 10^5^ events were acquired for each sample.

Cells	Passage	CD29	CD44	CD73	CD90	CD105	CD34	CD45
MSC#6	4	99.2%	99.6%	98.4%	96.2%	85.2%	9.9%	0.1%
T-MSC#6	(4 + 3)	99.0%	98.9%	96.2%	99.1%	82.3%	1.5%	0.4%
iMSC#6	(4 + 9 + 6)	99.2%	98.9%	98.0%	98.3%	81.0%	0.1%	0.0%
iMSC#6 (PD > 100)	(4 + 9 + 44)	98.2%	98.9%	97.1%	99.5%	73.8%	0.0%	0.0%
MSC#8	4	98.6%	99.4%	98.3%	98.9%	91.6%	9.8%	0.0%
T-MSC#8	(4 + 4)	93.9%	92.7%	86.3%	95.0%	46.0%	0.4%	0.1%
iMSC#8	(4 + 2 + 15)	99.9%	99.6%	99.6%	99.0%	42.1%	0.0%	0.1%
iMSC#8 (PD > 100)	(4 + 2 + 32)	98.8%	98.8%	98.7%	99.1%	38.4%	0.4%	0.2%
MSC#9	4	96.3%	97.0%	95.5%	96.2%	42.2%	0.6%	0.3%
T-MSC#9	(4 + 4)	98.4%	97.9%	69.6%	95.5%	71.7%	0.1%	0.2%
iMSC#9	(4 + 2 + 15)	98.4%	95.7%	97.3%	92.5%	37.3%	0.2%	0.4%
iMSC#9 (PD > 100)	(4 + 2 + 35)	98.8%	98.4%	98.7%	99.3%	44.6%	0.9%	0.9%
MSC#10	4	97.9%	97.5%	93.0%	98.7%	76.0%	0.6%	0.3%
T-MSC#10	(4 + 2)	98.5%	98.1%	85.4%	97.4%	73.7%	0.0%	1.0%
iMSC#10	(4 + 3 + 14)	98.9%	98.9%	92.8%	99.1%	74.0%	0.4%	0.2%
iMSC#10 (PD > 100)	(4 + 3 + 31)	98.4%	98.8%	93.9%	98.4%	82.4%	0.3%	2.7%
3a6	(*x* + 30)	99.2%	99.8%	98.0%	98.3%	61.7%	0.1%	0.0%

## Data Availability

The data used to support the findings of this study are available from the corresponding author upon request.

## Supplementary Materials

Supplementary Materials Supplementary Table S1: summary table showing the absorbance measurements in 3 different experiments (CCK8 1, 2, and 3 sheets) and the mean results from these experiments (data set sheet): before spinoculation (PRE-SPIN), after spinoculation (POST-SPIN), after three day-incubation with 0.5-5 mM VPA (POST-VPA), and after puromycin selection (POST-PURO).
